# Supplementary material for: Evaluating targeted COVID-19 vaccination strategies with agent-based modeling
Source: PLoS Comput Biol. 2024 May 31;20(5):e1012128. doi: 10.1371/journal.pcbi.1012128 (PMC11230632; doi:10.1371/journal.pcbi.1012128)
Supplement: Attachment — Submitted filename: review.pdf [file pcbi.1012128.s003.pdf]

Manuscript #: PCOMPBIOL-D-23-00170
Title: Evaluating targeted COVID-19 vaccination strategies with agent-based modeling

Summary:
The authors used a previously published agent-based model of SARS-CoV-2 transmission to compare how well four vaccine allocation strategies (Standard/Random, Risk-Based, Age-Based, and Ring) might succeed at reducing COVID-19 infections and deaths. These strategies were, and the authors considered these strategies in light of four vaccine supply levels and with/without a quarantine. Success was measured using cumulative incidence, cumulative incidence averted, and cumulative relative incidence averted ("effectiveness"). Simulations were performed for Marion County, Florida, United States, and were calibrated (particularly with respect to unmeasured behavioral variables) to fit existing time-series data. In terms of preventing infections, Ring Vaccination was the most effective while Age- and Risk-Based Vaccination were the least (but similar to each other). In terms of preventing deaths, this ranking was reversed. Quarantines and greater vaccine availability always prevented infections and deaths, in absolute terms. Relative to the Standard strategy, the differences between vaccination strategies were exaggerated by vaccine availability and altered by the time at which success was measured, but the ranking was unchanged. Finally, the authors concluded with a discussion of their results' generality, applicability, and limitations.

Recommendation:
This manuscript is in good shape. The authors have presented an impressive number of interesting scenarios and results in a fairly short text, relegating technical details to a bulky (but necessary) supplement. Because my suggested edits pertain to the text itself, rather than additional simulations or model details, I recommend that the authors be invited to resubmit the manuscript with **minor revisions**.

Comments:
1. Explain results about ring vaccination.
I was surprised to see that ring vaccination was simultaneously the best strategy for minimizing infections and the worst, at least in some settings, for minimizing deaths. Assuming this result is not due to some coding bug, there must be some intuitive explanation for this. I think the authors could do more to explain (a) how ring vaccination can be both good at preventing infections but bad at preventing deaths, and (b) why ring vaccination might be ineffective at preventing deaths. Is the answer something to do with the efficacy of the vaccine against infection versus severe disease? Is it because fewer shots are administered in ring vaccination, per Figure 4? To test the latter hypothesis, you could consider (in the supplement) one more vaccination strategy where whenever there are more vaccines than eligible vaccinees, the excess shots could be distributed randomly throughout the population. This analysis is not strictly necessary; however, additional text explaining this result would be helpful.

2. Extensions and limitations for other pathogens/epidemics.
The Discussion lays out how these results pertain to the COVID-19 pandemic in Florida, and is (understandably) cautious about extrapolating these results to other locations and scenarios. However, I am curious how general the results of this manuscript are. Do the authors expect random vaccination to prevent deaths better than ring vaccination for pathogens with different disease progressions or modes of transmission (e.g, vector-borne)? What about vaccines with different types of efficacy (e.g., against infection versus against hospitalization)? Rather than (or in addition to) speculating, the authors might also state some caveats the reader should bear in mind when considering applying these results in other contexts.

3. Clear definitions of key outputs.
Most of the paper was clearly written, but I would have preferred an explicit (e.g., mathematical) definition of "cumulative effectiveness" or "relative incidence averted," since this was one of the main outputs considered in the Results. The main text explains how to interpret effectiveness values, but as I read on I felt a concrete definition would have been helpful. There's an equation for this in Supplement S1, Page 31—could this be reproduced or referenced in the main text?

I was also confused by this sentence from the Abstract: "We also find that the magnitude of strategy effectiveness depends on when assessment occurs (e.g., after delta vs. after omicron variants)." This result became perfectly clear (and interesting) in Results on Page 15. For clarity, consider revising to "…depends on whether effectiveness was assessed after the alpha, delta, or omicron waves."

4. Figures.
Figures are nicely made and easily read. I had the following concerns.

- **Figure 1a** suggests that critical infection always leads to death in the model. Is this correct?
- In **Figure 2d**, wouldn't it make more sense for just the dashed lines between the blue people and the workplace to be grayed out, rather than the people themselves?
- It was not clear to me that the curves in **Figures 3b-d** were *user-defined* inputs selected through calibration, rather than data fed into the model. This is mentioned in the Figure 3 caption and commented on in Page 21, but should also be mentioned in the main text of the Methods.
- **Figure 7** is very helpful. Is each panel a slice of Figure 6? Is it possible to state this explicitly in the caption, e.g., that these are the values obtained from dates X, Y, Z in Figure 6?

5. Other comments:

A. The model includes a quarantine during calibration to actual case data from Marion County. What about other NPIs, such as masking? Is this effect combined with or eclipsed by the effect of a quarantine?
B. Do modeled individuals change their behaviors depending on their infection outcomes?

C. The paragraph at the end of Page 5 describes how the model structure gives rise to risk-groups within the population. This sounds like a feature of the model combined with an emergent property. It would be helpful to have a reference to either the Supplement or a past paper that expands on this property.
D. I slightly prefer Methods and Results to be written in the past (rather than present) tense. However, this is a stylistic preference, and both approaches are common among modeling papers.
