# Supplementary material for: Evaluating targeted COVID-19 vaccination strategies with agent-based modeling
Source: PLoS Comput Biol. 2024 May 31;20(5):e1012128. doi: 10.1371/journal.pcbi.1012128 (PMC11230632; doi:10.1371/journal.pcbi.1012128)
Supplement: Attachment — Submitted filename: PLOS_comp_bio_reviewer_response.pdf [file pcbi.1012128.s004.pdf]

Reviewer 1 Recommendation
This manuscript is in good shape. The authors have presented an impressive number of interesting scenarios and results in a fairly short text, relegating technical details to a bulky (but necessary) supplement. Because my suggested edits pertain to the text itself, rather than additional simulations or model details, I recommend that the authors be invited to resubmit the manuscript with minor revisions.

We appreciate the reviewer's useful feedback. Please see our responses below for how we have incorporated these items into the manuscript.

Reviewer 1 Notes
1. Explain results about ring vaccination.
- I was surprised to see that ring vaccination was simultaneously the best strategy for minimizing infections and the worst, at least in some settings, for minimizing deaths. Assuming this result is not due to some coding bug, there must be some intuitive explanation for this. I think the authors could do more to explain (a) how ring vaccination can be both good at preventing infections but bad at preventing deaths, and (b) why ring vaccination might be ineffective at preventing deaths. Is the answer something to do with the efficacy of the vaccine against infection versus severe disease? Is it because fewer shots are administered in ring vaccination, per Figure 4? To test the latter hypothesis, you could consider (in the supplement) one more vaccination strategy where whenever there are more vaccines than eligible vaccinees, the excess shots could be distributed randomly throughout the population. This analysis is not strictly necessary; however, additional text explaining this result would be helpful.

We agree that this result is counter-intuitive. The concise explanation is that ring-vaccination de facto *prioritizes young people who interact a lot, but are relatively unlikely to die, while neglecting seniors who are far more likely to have severe outcomes.* ***Fig S2 in SI2 (Results)*** *illustrates this disparate age-specific effect, showing that seniors (top row) are under-served by ring vaccination, while school-age children (bottom 2 rows) are the best served.*

We made additions to the Results and Discussion sections emphasizing this point. In ***Results, page 15****: "Conversely, the ring vaccination program has the effect of targeting people with many contacts, such as students and working age adults. As a result, ring vaccination tends to unintentionally prioritize people who are generally younger and healthier than average, and thus at lower risk of severe disease (see Figs S2 and S11 in S2 Additional Results)."*

In ***Discussion, page 19****: "Because ring vaccination targets high-transmission risk individuals, and they tend to be young and working age adults, this approach has the effect of shifting the limited vaccine supply away from relatively older individuals at increased risk of severe outcomes."*

2. Extensions and limitations for other pathogens/epidemics.

- The Discussion lays out how these results pertain to the COVID-19 pandemic in Florida, and is (understandably) cautious about extrapolating these results to other locations and scenarios. However, I am curious how general the results of this manuscript are. Do the authors expect random vaccination to prevent deaths better than ring vaccination for pathogens with different disease progressions or modes of transmission (e.g, vector-borne)? What about vaccines with different types of efficacy (e.g., against infection versus against hospitalization)? Rather than (or in addition to) speculating, the authors might also state some caveats the reader should bear in mind when considering applying these results in other contexts.

We agree with the reviewer that caution is warranted when generalizing not just in setting, but also with respect to the pathogen. We have revised the Discussion section to include this caveat about the results, with some modest speculation about the kind of features that would affect generalizing the results. Specifically, on ***page 21****: "The rankings of strategies are also pathogen- and, to a lesser extent, vaccine-specific. As such, future analyses of pandemic disease will need to repeat studies like this one to weigh the relative merits of different distribution programs. COVID-19 severity increases dramatically with age. This in turn increases the benefits of strategies that prioritize disease-risk, particularly in settings with relatively flat demographic distributions. Age is also a particularly easy, practical criterion to develop a strategy around. This severity profile is not a feature of all respiratory pathogens, however, let alone of pathogens generally. Additionally, SARS-CoV-2 is highly transmittable even in the absence of symptoms, which works against active response measures. Again, this feature is not ubiquitous among pathogens: even SARS-CoV-1 had a different-enough transmission profile for ring-based approaches to be effective"*

3. Clear definitions of key outputs.
   - Most of the paper was clearly written, but I would have preferred an explicit (e.g., mathematical) definition of "cumulative effectiveness" or "relative incidence averted," since this was one of the main outputs considered in the Results. The main text explains how to interpret effectiveness values, but as I read on I felt a concrete definition would have been helpful. There's an equation for this in Supplement S1, Page 31—could this be reproduced or referenced in the main text?

We have now incorporated the definition of cumulative effectiveness into the ***main text Methods (see Equation 1 on page 12)****.*

- I was also confused by this sentence from the Abstract: "We also find that the magnitude of strategy effectiveness depends on when assessment occurs (e.g., after delta vs. after omicron variants)." This result became perfectly clear (and interesting) in Results on Page 15. For clarity, consider revising to "...depends on whether effectiveness was assessed after the alpha, delta, or omicron waves."

*We agree this phrasing is more clear and have made this change **in the abstract**: "We also find that effectiveness of a strategy depends on whether effectiveness is assessed after the alpha, delta, or omicron wave."*

4. Figures.

- Figure 1a suggests that critical infection always leads to death in the model. Is this correct?

*Thank you for catching this error; the figure was wrong. Critical infections can revert to less severe stages and then recover. We have revised **Figure 1** and its caption to clarify this as follows: "I_C individuals can either die or revert to I_S, in which case they will eventually recover."*

- In Figure 2d, wouldn't it make more sense for just the dashed lines between the blue people and the workplace to be grayed out, rather than the people themselves?

*In Figure 2, panels B and D purposefully differ in how elements are grayed out. In Panel B, we only gray the arrows because the locations that are no longer connected still exist and have transmission but the people of this highlighted household merely choose not to interact with these locations. Contrary to this, in Panel D, patrons that would normally interact with this workplace are no longer there and thus are grayed out. We have clarified this distinction in the caption of **Figure 2**: "When perceived risk is high, risk-intolerant (blue) employees of high-transmission-hazard workplaces still go to work, while risk-intolerant patrons stop visiting these locations (and thus are grayed-out)."*

- It was not clear to me that the curves in Figures 3b-d were user-defined inputs selected through calibration, rather than data fed into the model. This is mentioned in the Figure 3 caption and commented on in Page 21, but should also be mentioned in the main text of the Methods.

*We have clarified this in the Methods, in the caption of **Figure 3**, and by slightly reordering the panels. Now the first four panels are inputs. The seasonality (a) and detection curves (b) are user defined. Vaccine coverage (c) is a scenario input, but defined by empirical summaries of different settings; the vaccine coverage in **Figure 3c** represents the actual Florida vaccine rollout, which is the coverage used for model fitting. The societal-risk perception curve (d) ends up being an input, but is determined by iteratively accounting for residuals in the focal outputs. Panels (e-i) are all comparisons to Florida surveillance measures.*

- Figure 7 is very helpful. Is each panel a slice of Figure 6? Is it possible to state this explicitly in the caption, e.g., that these are the values obtained from dates X, Y, Z in Figure 6?

*The reviewer is correct. As suggested, we have revised the caption of **Figure 7** to make this point clearer, adding the specific dates as follows: "these are effectively 'snapshot' values of the*

[Fig 6] curves at 2021-05-07 (post alpha), 2021-11-26 (post delta), and 2022-03-07 (post omicron)"

5. Other comments:

- The model includes a quarantine during calibration to actual case data from Marion County. What about other NPIs, such as masking? Is this effect combined with or eclipsed by the effect of a quarantine?

*We recognize that other personal-protective behaviors (PPB; e.g. masking, physical distancing, etc.) are important in controlling transmission. However, with limited (or absent) empirical data, these behaviors were challenging to explicitly incorporate. We are reading the reviewer's reference to "quarantining" here to refer to the "lockdown" period undertaken in Florida, which we included during calibration, and not isolation of known case contacts. The latter was in some intervention scenarios, but not included in calibration of the Florida model, as that was not policy. PPBs in our model are distinct from both of these. Essentially, when we calibrate the model we put in known interventions with a clear model interpretation (alongside other empirical mechanisms), and fit other parameters, including the reactive PPB model, which covers the full time span of the model, and applies alongside all other measures. As we have addressed in the discussion, our approach has limitations, including that the risk perception would likely change in response to changes in the package of response activities. We have revised the description of this in **Section 5.4 of SI1 (Methods)**: "Individuals during the pandemic made a wide range of behavioral changes to reduce their chance of SARS-CoV-2 infection, including masking, physical distancing, hand-washing, switching to outdoor activities, etc. Adoption of these measures was heterogeneous across populations, changed over time, and varied in effectiveness. Parameterizing a personal protective behavior (PPB) model directly from data has proven difficult, but omitting behavioral changes altogether, in our experience, has made it impossible to reproduce COVID-19 trends. Therefore, due to the lack of available data on individual behaviors, we opted to represent behavior in a more general, high-level way."*

- Do modeled individuals change their behaviors depending on their infection outcomes?

*In our model, individuals will refrain from attending work or school (if applicable) if they are infected and are experiencing severe or critical symptoms. This has been clarified in **Section 5.4 of SI1 (Methods)**: "Lastly, in addition to these behavior changes, infected individuals will refrain from attending work or school (if applicable) when they are experiencing severe or critical symptoms."*

- The paragraph at the end of Page 5 describes how the model structure gives rise to risk-groups within the population. This sounds like a feature of the model combined with an emergent property. It would be helpful to have a reference to either the Supplement or a past paper that expands on this property.

We recently submitted a manuscript (https://doi.org/10.48550/arXiv.2306.11003) explaining the issue as follows: "Many of the policy questions we faced concerned specific groups of people,

either for their characteristics (e.g., age) or for their activities (e.g., where they worked). These groups are not uniformly distributed among the population, but instead tend to mix heterogeneously…. The sort of heterogeneity arising from population age structure and contact dynamics are largely accounted for by simply creating our synthetic populations based on real-world data as much as possible." As requested, we now cite this paper (Ref 16), in the ***main text on page 6****.*

- I slightly prefer Methods and Results to be written in the past (rather than present) tense. However, this is a stylistic preference, and both approaches are common among modeling papers.

We appreciate the reviewer's thoughts on this, but to avoid potentially introducing grammatical errors, we are not making this change at this time. We will implement any style guidance that the journal copy editors direct.

Reviewer 2 Recommendation
The manuscript is clear, and the model seems well designed and well documented. The following comments aim to clarify aspects of the methodology and results.

Reviewer 2 Notes
1. Manuscript:

- Fig. 3: I'm not sure mixing linear and log scales is especially helpful here...I was greatly confused why the omicron peak wasn't larger until I noticed this (and if the y-axis labels could be made darker, that would help).
- Fig. 3: The fit is impressive. That said, I'd be curious to know if seasonal forcing was really required to get a good fit (no need to rerun the calibration though, I'm sure it was an extremely involved process!).

Having considered both linear and log versions of these figures, we believe there are important features that are more easily extracted from the log versions, but we appreciate that readers may anticipate linear versions. We have modified Figure 3 to improve the labeling, including explicitly stating which panels have log scales.

The close alignment of results is in part attributable to using a time-varying societal risk perception. While substantially more constrained than simply varying an overall transmission parameter, it is in a big picture sense eliminating the residuals. We introduced seasonal forcing into the model prior to doing the societal risk perception adjustment, and did not consider removing it once we embarked on fitting that effect; there is prior evidence to suggest that coronaviruses experience substantial seasonal forcing (https://doi.org/10.4414/smw.2020.20224)*. While we suspect that the model could still achieve good alignment without a seasonal factor, we expect that the emergent pattern in risk perception trend would no longer match our own experience of behavior in Florida, or that we would have to modify other parameters to substantially mismatch their intended meaning and the available empirical evidence.*

- p. 9: Given the wide variability of vaccination rates between countries (https://www.thenationshealth.org/content/51/8/1.3), and especially between lower- and upper-middle income countries (https://data.undp.org/vaccine-equity/), the authors are encouraged to use specific country archetypes rather than lumping all LICs, MICs, etc.

We appreciate the reviewer's point, but feel that this specific proposed change would lead readers to draw inappropriate conclusions from the work. We aim to show what effect vaccine supply has on strategy performance, not to provide results for specific countries that the model is not closely calibrated to. That said, we agree with the reviewer that there is substantial diversity in national outcomes within broad categories like LIC and MIC, and as such it is inappropriate to suggest those average results apply to all members of the category. We have made several revisions throughout the manuscript and figures to reflect this change. Briefly, we now refer to the different scenarios as the low, middle, and high supply scenarios (LS, MS, and

HS). Where we define these scenarios, however, we sometimes still refer to lower-, middle-, and high-income countries, as those designations are what we used to develop these scenarios.

- p. 12: I don't see anything about the delay between the vaccination event and the immune response. In practice this takes considerable time (days to weeks). While it is less important on a population level, wouldn't this delay need to be taken into account in order to have accurate ring vaccination results?

Thank you for identifying this omission from the methods. The reviewer is correct that vaccination requires time to induce an immune response, and that this delay is critical to consider when modeling ring vaccination. The model does in fact have a delay from vaccination to protection. We have clarified this in the ***main text (page 12)****: "Vaccine efficacy begins 10 days after vaccination." We have also repeated this clarification in* ***Section 2.2.2 in SI1 (Methods)****: "This immune protection takes effect 10 days after a vaccine dose is received."*

- Fig. 5: The labels are very confusing; it's easy (for me) to forget that "LIC" refers *only* to the amount of vaccine provided. Less "loaded" labels such as "Low vaccine coverage" are probably more appropriate.

We have made this adjustment as part of a broader relabeling revision described above; we now refer to supply levels, rather than country categories.

- Fig. 5: The different strategies are too similar to tell apart on this graph. Why not make this into two graphs, one for different vaccine coverage levels and the standard program, the other for the different strategies for USA vaccine coverage?

*That proposal would not actually fix the relative scaling; the HS and USA programs are very similar. We attempted a few other approaches, including log scaling and splitting LS/IS vs HS/USA, and did not find that those resolved the problem either. As indicated in the discussion, we believe the most reliable output of the analysis is ranking (**Figure 7**), followed by effectiveness (**Figure 6**), then absolute outcomes (**Figure 5**). The fact that they are difficult to distinguish in **Figure 5** is informative in its own way: for absolute outcomes, strategy doesn't matter that much in the higher supply scenarios. Policy makers thus should choose the easiest/cheapest option.*

- p. 20: "Our quantitative results for incidence of infections and deaths should not be interpreted as estimates for the LIC and MIC settings" -- I agree, to the point that I think these labels are doing more harm than good, even if qualified in the discussion as they are here.

We have fixed this issue as part of a broader relabeling revision, described above.

- p. 21: "we are comfortable with this approach and suspect that attempts to compare to relevant empirical data (e.g., risk surveys, changes in mobility patterns) would be

consistent." -- it seems odd that the authors would speculate on this when mobility data is available for Florida (Facebook, Google, SafeGraph, etc). Why not actually do this comparison? One would not expect there to be a perfect correlation, but if there's no correlation, that would suggest, at minimum, an alternative interpretation to "societal risk perception" as an overall transmissibility modifier.

After revisiting this topic, we feel we should have treated it more carefully. We have rewritten this paragraph in the ***discussion (on pages 21–22)*** *to provide a better explanation of what we do and don't know about appropriately modeling reactive behaviors:*

"Additionally, our model of risk perception and personal protective behaviors is essentially accounting for model residuals. When comparing the trends in inferred perceived societal risk to epidemic trends in our reference population, we see perceived risk slightly lagging incidence dynamics, with gradually dampening reactions. This matches our intuitions about how populations actually reacted to pandemic waves, but this argument is largely anecdotal. Early in the pandemic, mobility data appeared sufficient to characterize behavior shifts, but we found this relationship diverged within a few months, plausibly due to the myriad social and business adaptations that occurred. More empirical studies are needed to identify what additional measures should inform transmission parameters beyond changes in mobility trends. Such work would both reduce the need for ad hoc residual adjustments and improve the portability of a behavior model. However, even these additional data would not necessarily address how behavior changes with prevailing pandemic conditions. That remains an important open question in infectious disease modeling, which needs focused work to inform what data to collect to appropriately parameterize explicit reaction models. We did not attempt to codify our approach to the behavior model in a way that would respond to prevailing conditions. Instead, we simply use the risk curves from the fitting stage for the alternative strategy scenarios. Those scenarios result in different incidence, which in real populations would likely affect risk perception and thus behavior."

2. Method supplement:

- The authors are thanked for their comprehensive supplement and open-source code.
- p. 1: How can there be 33,800 workplaces and only 118 schools? This implies there are almost 300 workplaces for every school, which doesn't seem right.

While we also found this ratio surprising when the reviewer pointed it out, our validation of data sources suggests that it is reasonable. Using the Florida Department of State – Division of Corporations as a secondary empirical source for workplaces, we confirmed that the dataset we used contains a reasonable number of total workplaces. This secondary source estimates approximately 2.5 million workplaces statewide in May 2020 while our primary source, the National Corporation Directory (which also includes street addresses and NAICS business classifications) estimates approximately 2.3 million. With this secondary source, we compared the ratio of workplaces at the state level to the synthetic population used in this work. Comparing the number of workplaces from the Florida Department of State (~2.5 million) to the number of schools statewide from the National Center for Education Statistics (7639), we find a

workplace-to-school ratio of ~327. Performing this same comparison using our Marion-county-like synthetic population (with 33826 workplaces and 118 schools) returns a workplace-to-school ratio of ~287. We find that these ratios are consistent with one another. There are some aspects of the data that may aid to explain why there are more workplaces than one may expect in our synthetic population. Approximately 91% of workplaces are not patronized, and about 85% of workplaces have only 1–2 employees. Combining these filters, we find that ~77% of workplaces have 1–2 employees and are not patronized.

- It sounds like the model doesn't include waning immunity, except for the 60-day box function. This is unlikely to capture realistic immune dynamics, which show continuous, long-term exponential decay.

We agree; the manuscript correctly detailed the immunity assumptions we were making, but having immune waning turned off was unintentional. We have now corrected this oversight. As a consequence, we have needed to recalibrate the model. We have been able to simplify some assumptions, as well as reproduce an additional empirical observation, namely that seroprevalence is higher in children than in the general population. We have explained the details of our waning sub-model in ***Section 2.2.3 in SI1 (Methods)****.*

- Table S5: Are these efficacies, or risk ratios...? Does an efficacy of 1.0 imply perfect protection or no protection? I assume the former because they increase from doses 1-3, but doesn't VE_H = 1.0 imply that no one who is doubly vaccinated would die? I am confused.

Table S6 (previously Table S5) shows efficacies. An efficacy of 1.0 does imply perfect protection against an outcome. So for the VE_H values the reviewer highlights, yes, the model is assuming absolute protection from death; this is consistent with estimates in the literature (see [21] in the Supplement, available here: https://www.medrxiv.org/content/10.1101/2021.05.20.21257461v2.full.pdf). For the infection efficacy, the protection represents a baseline and is modified by various factors, including waning and VOC immune escape.

- Table S6: Where do these values come from?

We now clarify that these values come from the manual calibration process in the caption of ***Table S7*** *(formerly S6)* ***in SI1 (Methods)****: "These are assumed values, chosen during model calibration to reproduce age-specific seroprevalence and mortality while staying as simple as possible"*

- Eq. S5: Why is viral shedding discretized into high and low shedding instead of being drawn from a distribution? Does the calculation of the Pareto distribution of infections take into account the fact that some people have many more contacts than others? (i.e. superspreaders both shed a lot and have above-average numbers of contacts.)

Creating two shedding categories rather than sampling from a continuous distribution is merely a simplifying assumption. This has been clarified in ***Section 5 of SI1 (Methods) on page 23****: "This viral load calculation is performed on a per-infection basis, and is only dependent on whether the infection will ever be symptomatic."*

- Table S7: Why would there not be a fixed probability of contact rather than a fixed number? It doesn't make sense to draw more contacts in a workplace, say, than there are people in that workplace. Where does the assumption of 70% come from?

(Now Table S8) This is related to the distinction between density versus frequency dependent interactions. Because we assume workplace interactions are frequency dependent–i.e., the employees interact with a fixed number of individuals, independent of business size–we assume a fixed number of traced contacts. We do censor the contacts based on business size; we have modified the S8 caption text to explicitly state this: "If a given location has fewer available contacts than the sampled value, the number of available contacts is used instead."

For the 70% contact tracing rate: this an optimistic-but-imperfect assumption for the purposes of the scenario analysis. Since contact tracing in our model is used only in the context of ring vaccination (which did not occur in Florida), this assumption does not impact our fitting.
