# Supplementary material for: Evaluating targeted COVID-19 vaccination strategies with agent-based modeling
Source: PLoS Comput Biol. 2024 May 31;20(5):e1012128. doi: 10.1371/journal.pcbi.1012128 (PMC11230632; doi:10.1371/journal.pcbi.1012128)

Seasonal forcing

I still have concerns here (and the paper the authors cite isn't on SARS-CoV-2; it's from very early in the pandemic, extrapolating from other coronaviruses). To my knowledge, no study has found conclusive proof of seasonality, although several studies have been suggestive. What data does exist seems to suggest a 12-month seasonal forcing term: see e.g. https://www.medrxiv.org/content/10.1101/2022.01.26.22269905v2.full-text. So now I am especially confused why the authors chose a 6-month term here. I can't find any explanation of the seasonality parameter in either the main text or the supplement. This aspect of the model either needs much more explanation and justification, or should be removed.

We have now considered a non-seasonal model. In summary, seasonality assumptions do not affect the conclusions of our paper. However, as seasonality is common across a wide range of respiratory pathogens, we still prefer a model with seasonality. We have revised the explanation and justification of that choice in the main text and supplement, with further discussion below.

For this revision, we have rerun the fitting and scenario analysis both with and without seasonal forcing. The results are not quantitatively identical, but the differences are very minor and qualitative conclusions about the preferred vaccine programs do not change. While our particular seasonality model may not generalize to other locales, the non-seasonal results indicate that, as expected, those assumptions do not seem to affect these kinds of decisions. We have reproduced the updated main text figure 7 here (with seasonal forcing), as well as the complementary figure (without seasonal forcing) from the supplement; they are practically indistinguishable:

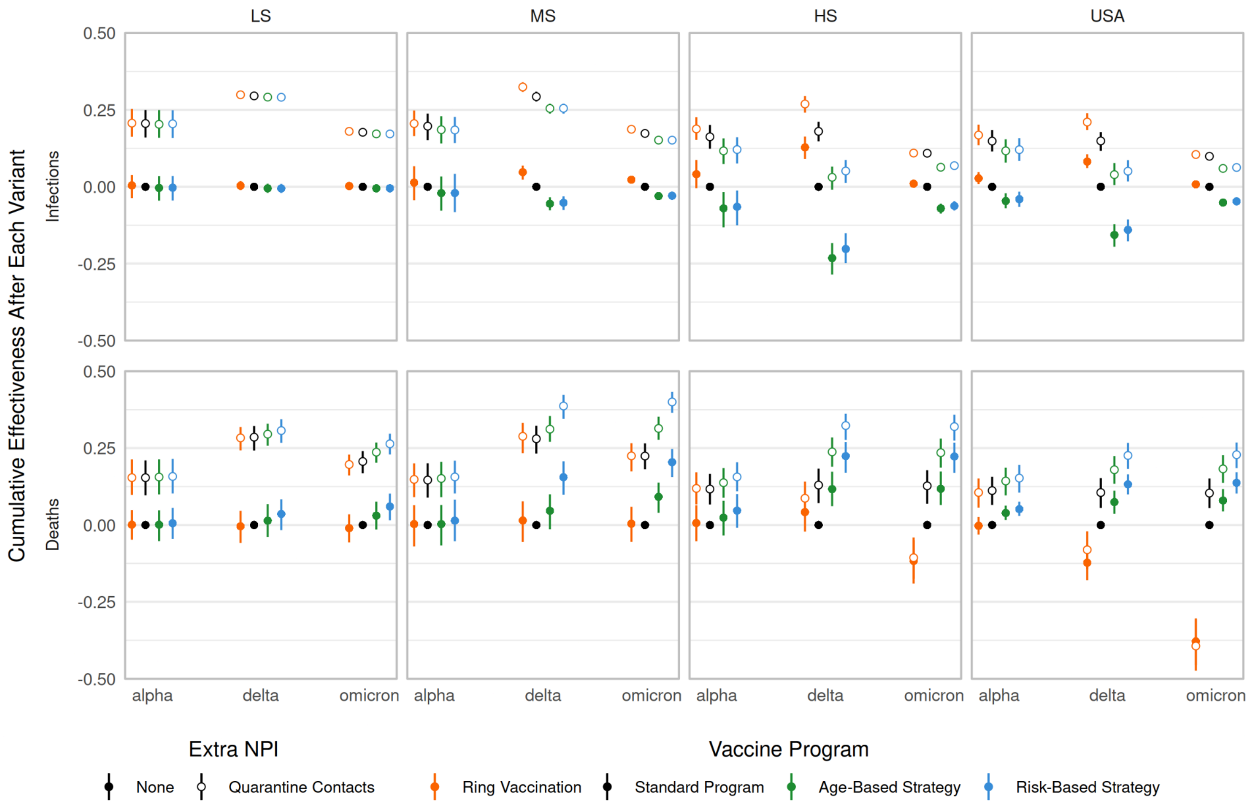
LS
MS
HS
USA
Cumulative Effectiveness After Each Variant
Infections
Deaths
0.50
0.25
0.00
-0.25
-0.50
alpha
delta
omicron
Extra NPI
None
Quarantine Contacts
Vaccine Program
Ring Vaccination
Standard Program
Age-Based Strategy
Risk-Based Strategy

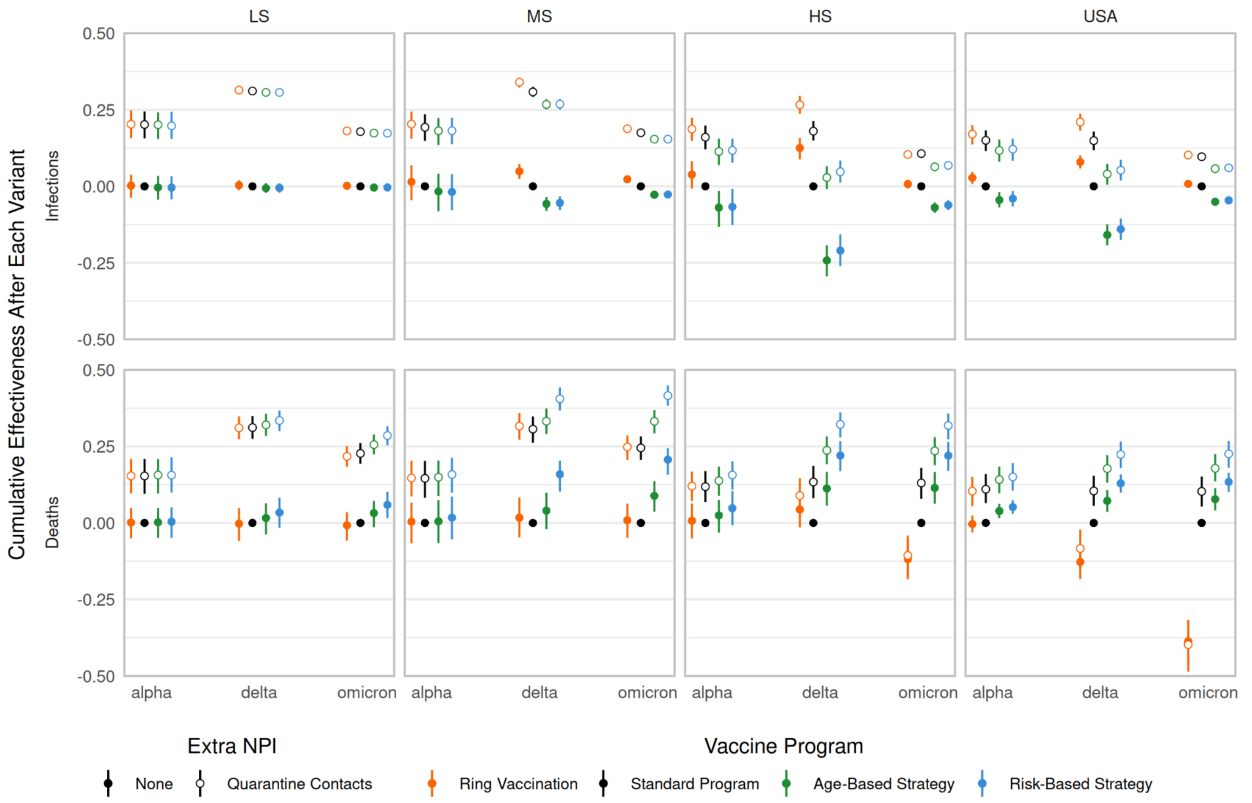
LS
MS
HS
USA
Cumulative Effectiveness After Each Variant
Infections
Deaths
0.50
0.25
0.00
-0.25
-0.50
alpha
delta
omicron
Extra NPI
None
Quarantine Contacts
Vaccine Program
Ring Vaccination
Standard Program
Age-Based Strategy
Risk-Based Strategy

Regarding justification for our focus on the model with seasonal forcing: respiratory pathogens broadly exhibit seasonal patterns, which appear to be a mix of direct environmental factors on the pathogen or transmission physics, as well as changes in human behavior patterns in response to seasonal factors which change transmission opportunities [Fisman, Clinical Microbiology and Infection, 2012, https://doi.org/10.1111/j.1469-0691.2012.03968.x], though the exact mixture of mechanisms behind observed seasonal trends remains an open question. We agree with the reviewer's interpretations about a 12-month seasonal pattern in the suggested paper, but that conclusion is driven by more northern localities in US (Denver and New York) and Europe (Netherlands and Northern Europe). The seasonality of SARS-CoV-2 in the tropical and subtropical regions in the study, such as Nakhon Si Thammarat, Thailand, Hong Kong, and Guangzhou are markedly different. Our focal setting, Florida, is more environmentally consistent with the latter countries. Susswein et al (eLife, 2023, https://doi.org/10.7554/eLife.80466) found differing seasonality between regions of the U.S., with some evidence of biannual seasonality in the South. We expect that even within the southern U.S., seasonality varies substantially.

Our usage of a 6-month seasonality model is rooted in the observation that Florida has experienced summer and winter incidence peaks each year of the pandemic. We have added a figure highlighting this to the supplement for the three years we model (Fig. S9 reproduced below, showing weekly deaths normalized by the total deaths that year).

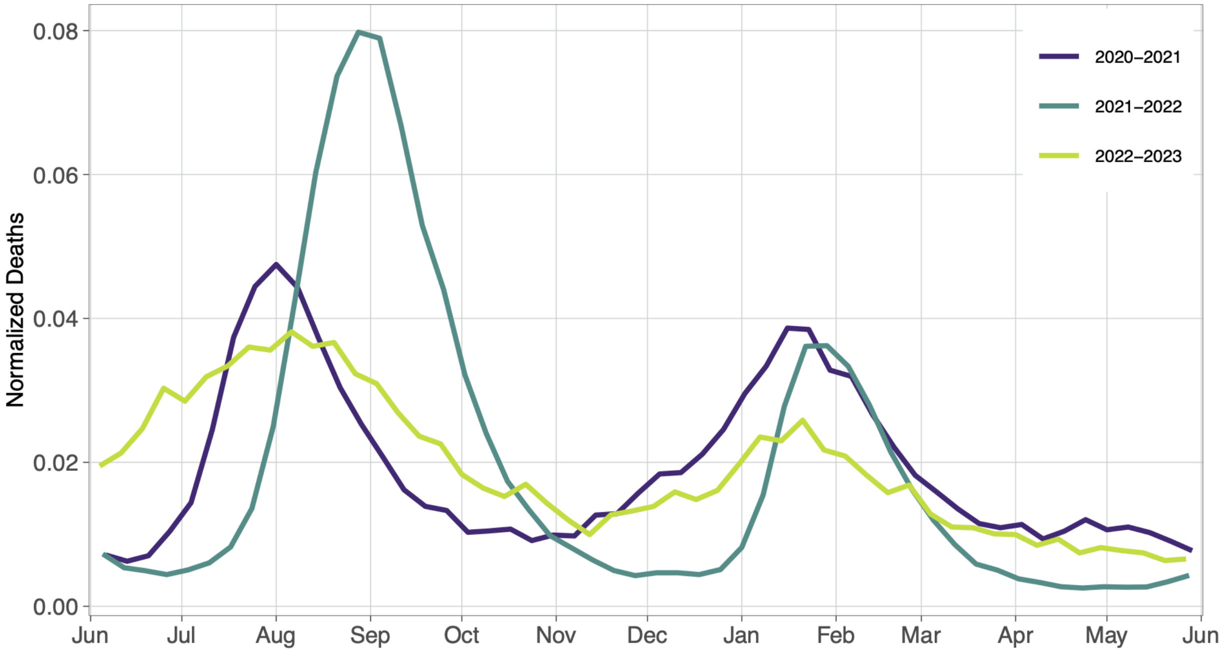

We believe this seasonality arises because both summer and winter weather extremes in Florida tend to push people towards indoor activities; this is reflected in mobility data (Susswein et al), tourism indices such as those produced by companies like Weather Spark (intended to quantify how "nice" the weather is; see new SI 1 Fig S10, reproduced below), and consistent with our first-hand experiences.

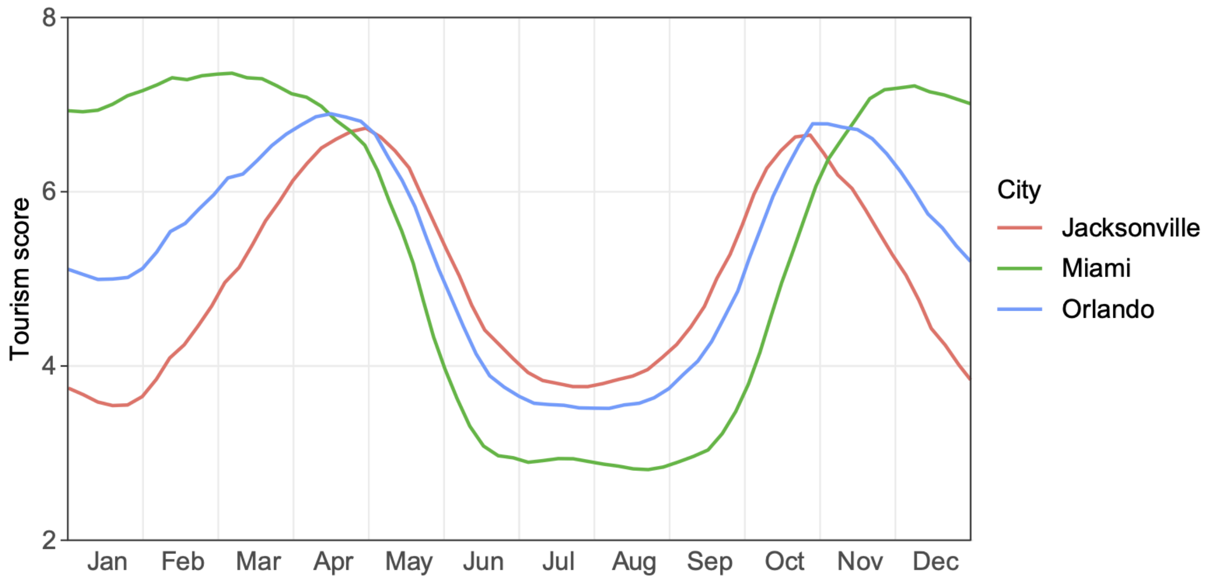

We have updated our discussion and supplement to expand discussion of seasonality and incorporate additional results. We have clarified that our use of Neher et al. 2020 is only to inform the amplitude parameter in the seasonality model.

LIC/LMIC

The authors are thanked for making this change, but are asked to remove all remaining references to income levels (such as in the abstract). According to Our World in Data (https://ourworldindata.org/covid-vaccinations), the top 5 countries for vaccine doses per 100 people are Cuba, Chile, Japan, Brunei, and Cambodia. Needless to say, those do not fit into a neat income classification!

We agree with what we think the reviewer's intent here is–namely, that we should not suggest either our model scenarios or results are representative of particular countries. We have made further revisions in that direction, including removing reference to income levels in the abstract. The only remaining mention is in the methods section.

Strictly removing any mention of income categories, however, would obfuscate what we did methodologically. We do not think such an obfuscation represents good practice: we developed rough supply categories based on World Bank Income setting averages, and should plainly say so. We also think that, unfortunately, those income categories are informative, even if there is variability within them; per the UNICEF data we use (figure not included in manuscript):

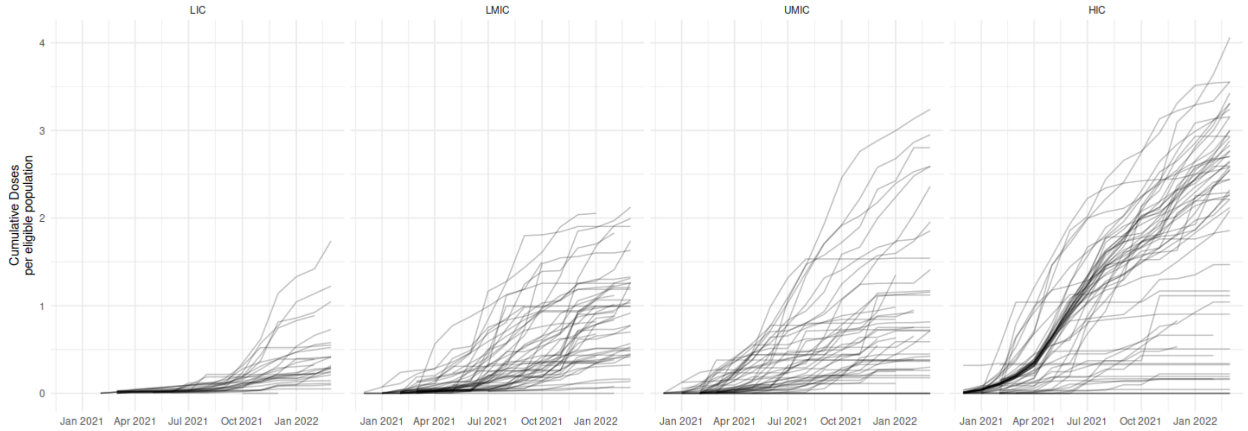

Results Fig. 5

Another way of phrasing my original comment is that there are 8 panels, each showing 8 scenarios: so 64 time series in total. This is fine for a supplement but seems like a lot for a paper. I want to ask: what's the bottom line? If the bottom line is, as the authors state, "strategy doesn't matter that much", there are probably simpler ways of making that point! I would much prefer seeing a handful of time series, and then a scalar metric (e.g. bar plot of final time points) for the full 8x4x2 matrix.

If we somehow indicated at some point that we think strategy doesn't matter, we apologize for the miscommunication. We agree the figure does not convey well what we think is the most important takeaway. We have therefore created a new visualization showing that there are clear strategy preferences. For reference, the new main text Figure 6 is reproduced here:

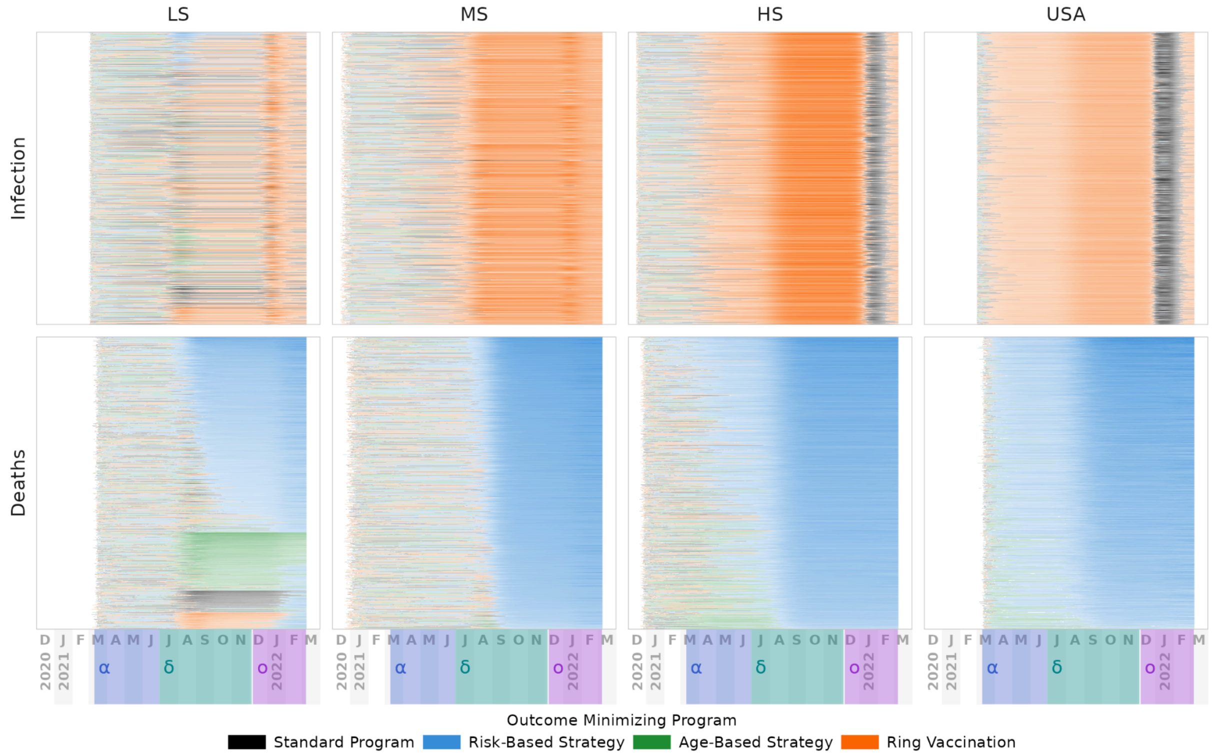

As main text Figure 7 (not reproduced here) shows, the magnitude also varies in a discernable way. It is beyond the scope of this work, however, to evaluate whether those differences are large enough compared to competing demands, for example in terms of cost of the inputs and relative value of the change in outcomes specific to the setting.

Mobility data

The additional clarification is appreciated. However, looking more closely at the "societal risk perception", I am having a hard time reconciling it with my experience. Especially in a state like Florida, I would've expected minimal changes in behavior after the initial wave of COVID. This is backed up by the mobility data (see e.g. https://ourworldindata.org/covid-google-mobility-trends) and face mask data (https://bmcpublichealth.biomedcentral.com/articles/10.1186/s12889-021-12175-9). In contrast, the "societal risk perception" seems to fluctuate from 0.5 to near-zero almost exactly every 4 months. I didn't quite follow Sec 5.4 of the supplement, but it sounds like the proportion of households that use "PPBs" are directly proportional to the current "societal risk perception", which would imply large month-to-month fluctuations in mask use. This doesn't seem to be supported by the data. The authors refer to "societal risk perception" as a "residual" in the model, and this seems like a potentially more correct explanation -- that there is a structural issue with the model that requires each wave to be artificially dampened. Perhaps mixing is too homogeneous? Perhaps immunity is waning too quickly? The authors are strongly encouraged to revisit their model assumptions and see if the magnitude of this "residual" can be reduced.

While mobility data initially held a great deal of promise, the usefulness of those measures as a predictor for outcomes rapidly degraded. While the reviewer is correctly reading those particular metrics, careful analysis of that kind of data has consistently shown it's just not that useful an indicator (e.g.: Badr, 2020, Lancet Infectious Disease, 10.1016/S1473-3099(20)30861-6; Kishore, 2021, Lancet Digital Health, 10.1016/S2589-7500(21)00214-4).

We would also prefer to replace a residual with an input trend. Finding and measuring such a trend, however, is an enormous project beyond the scope of this work. What is clear, however, is that mobility trends alone are not such a measure.

Workplaces

The issue is with equating "corporation" with "workplace". This is simply not valid. The extreme example is Delaware, with roughly two corporations for every adult and child in the entire state (1.9m corporations vs 1.0m people; see https://corp.delaware.gov/stats/). Removing "non-patronized" workplaces would go a long way towards addressing this (although ~30 workplaces per school still seems like too many workplaces to me!). What are the transmission implications of a workplace with a single employee -- none, right?

We share the reviewer's concern here with establishing a good empirical basis for building up workplace structure. While Florida is not as pathological on single-person businesses as Delaware, and there are real single-person, work-from-home businesses, we agree that on balance it is preferable to assume the single-person non-patronized businesses are not included in the distribution. When we apply this assumption, the distributions now look as below (this figure not included in the manuscript):

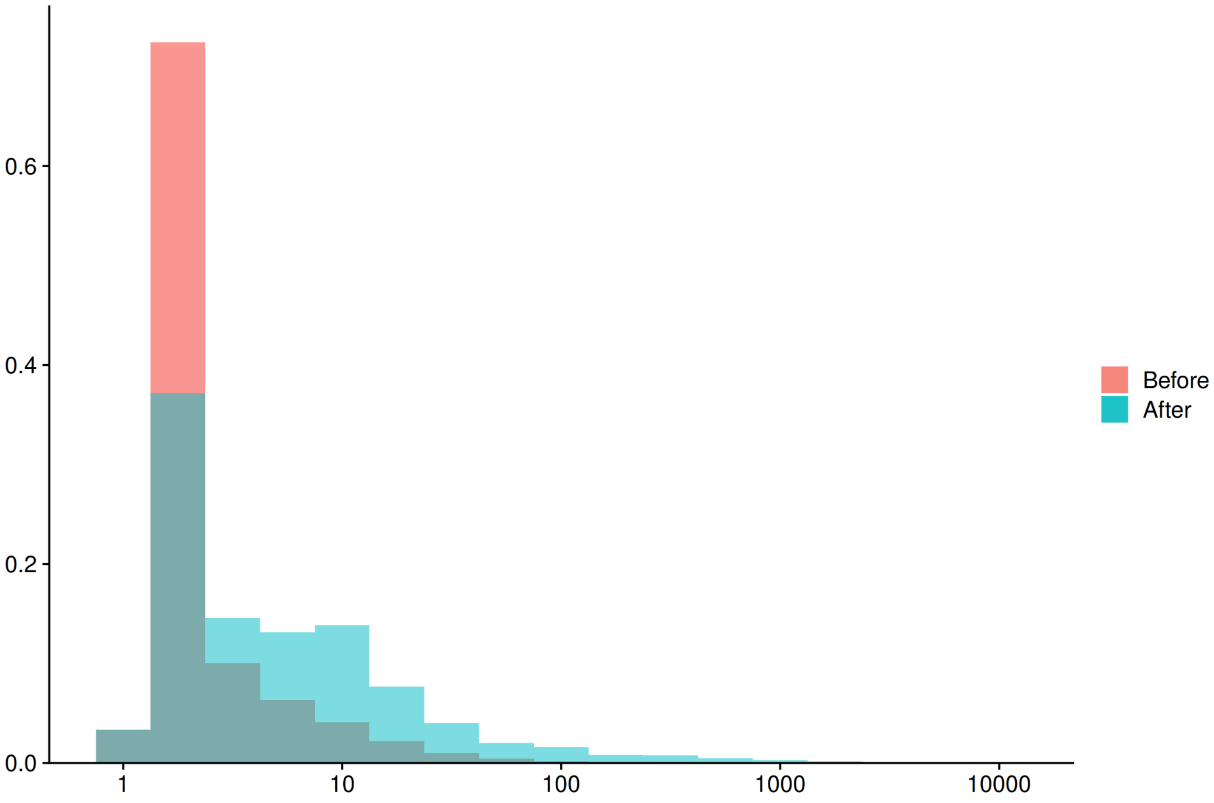
0.6
0.4
0.2
0.0
1
10
100
1000
10000
Before
After

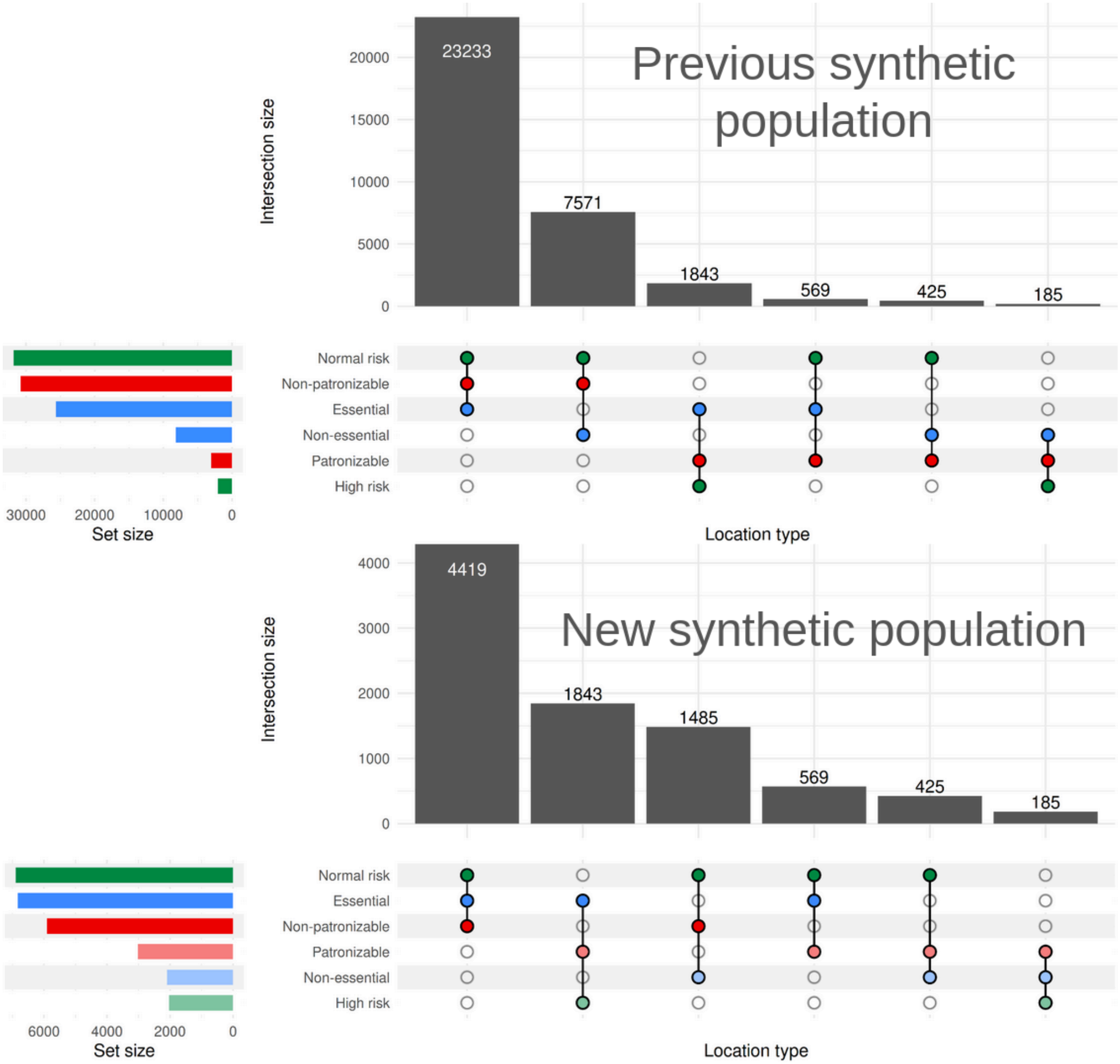

However, the change in workplace structure had little impact on quantitative outcomes, and none on ranking of strategies. These changes might be more consequential if we were evaluating work-place-based interventions.

Given that the number of workers in the model is constrained by census data, the transmission implication for unpatronized, single-employee workplaces is that those people are not transmitting at work, as they may be when they are instead placed in multi-employee workplaces. There may therefore be a modest shift in where transmission is happening which, again, might be more consequential if we were evaluating work-place interventions.

Vaccine efficacy

The citation given (https://www.medrxiv.org/content/10.1101/2021.05.20.21257461v2.full.pdf) was published only a couple months after the vaccines first became available. There are much better sources available now. More recent estimates put the efficacy at something closer to 60% (https://jamanetwork.com/journals/jamanetworkopen/fullarticle/2802473). It is essential to update this parameter, as the findings will likely change significantly.

The reference the reviewer cites pertains to vaccine performance against various levels of symptomatic disease (rather than infection) during BA.4 and BA.5 transmission, which emerged after the time period we model. Direct comparisons to our model inputs are therefore not straightforward. That said, the efficacy estimates provided by that paper seem consistent with our assumptions. The primary issue is that we were not sufficiently clear in our previous manuscript about how our VE model works, as we required the reader to check multiple tables to calculate strain-specific VE. The VE against susceptibility specified in Table S6 in the Methods Supplement is for baseline performance, and is reduced variant-specific immune escape, as shown in the new Fig S3 in the Methods Supplement, also provided below. This peak benefit also degrades according to a waning immunity model (consistent with results in work the reviewer references).

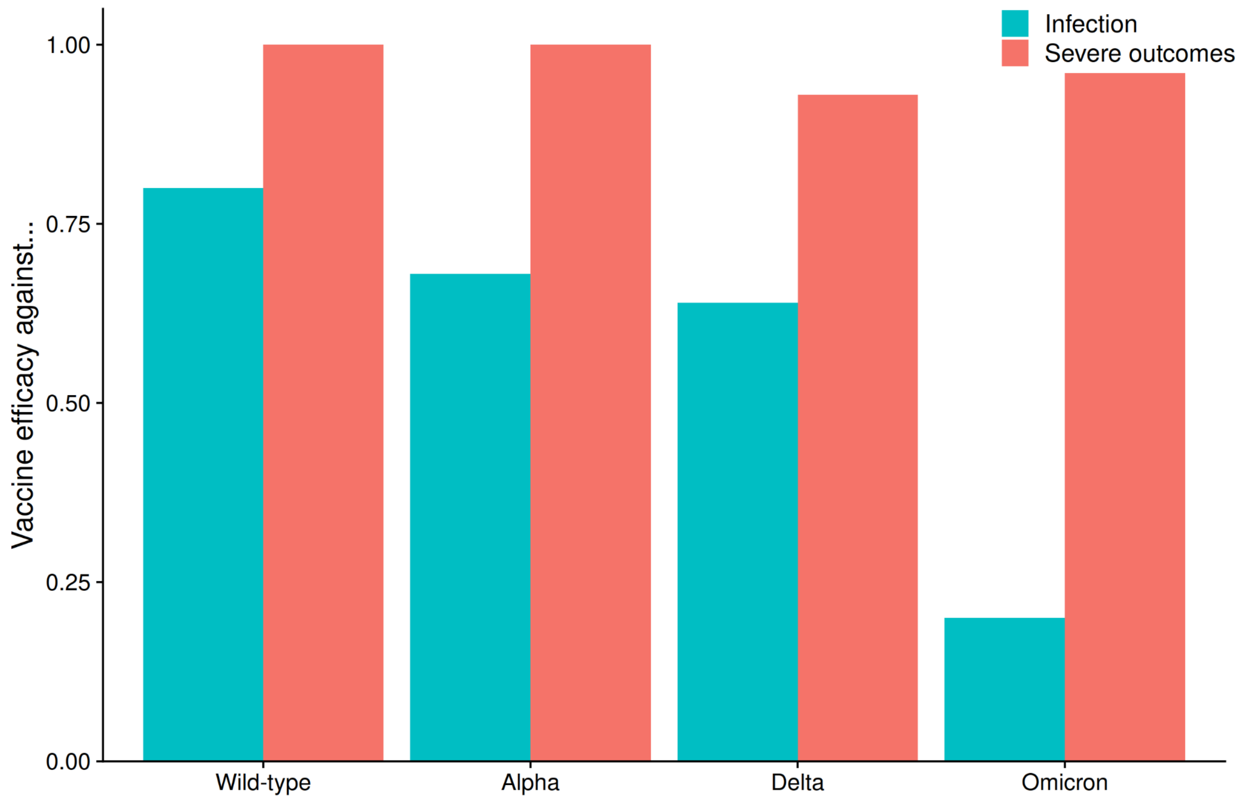

Supplement: Attachment — Submitted filename: PLOS_comp_bio_reviewer_response2.pdf [file pcbi.1012128.s005.pdf]
